# Lipid desaturation and cellular viability: mechanisms, stem cell insights, and a desaturation window model

**DOI:** 10.1016/j.jlr.2026.101036

**Published:** 2026-04-09

**Authors:** Keerthana Mohan, James M. Ntambi, Arieh Moussaieff

**Affiliations:** 1 The Faculty of Medicine, The Hebrew University of Jerusalem, Jerusalem, Israel; 2 Department of Biochemistry, University of Wisconsin-Madison, Madison, WI, USA; 3 Department of Nutritional Sciences, University of Wisconsin-Madison, Madison, WI, USA

**Keywords:** fatty acid desaturation, stearoyl-CoA desaturase (SCD), stem cells, differentiation, apoptosis, cell death, membrane fluidity, ER stress, cancer cell metabolism

## Abstract

Fatty acid (FA) desaturation is a key determinant of membrane physicochemical properties and influences multiple aspects of cellular viability and stress responses. A substantial body of evidence indicates that certain cancer cells exhibit heightened sensitivity to perturbations in FA desaturation, a feature that is also observed in cells with high differentiation potential. This sensitivity has been linked to changes in membrane composition, endoplasmic reticulum (ER) homeostasis, and signaling pathways. Insights from stem cell systems highlight the cell-type-specific nature of these processes. In particular, trophoblast stem cells (TSCs), which exhibit high monounsaturated fatty acid (MUFA) abundance, display opposite dependencies on MUFAs and express a distinct variant of stearoyl-CoA desaturase (SCD), compared with embryonic stem cells (ESCs), which are characterized by lower MUFA levels, suggesting that optimal MUFA to saturated fatty acid (SFA) ratios are required in a cell-type-specific manner. In this review, we synthesize current knowledge on the molecular and biophysical mechanisms linking FA desaturation to cellular viability, including its effects on membrane fluidity, protein function, and signaling pathways. Where stem cell-specific mechanistic data are limited, we draw on broader cellular systems to inform these mechanisms. We propose a “desaturation window” model, whereby deviations in either direction: excess saturation or insufficient saturation, disrupt membrane homeostasis and compromise cell survival. A clearer understanding of the mechanisms governing cell viability in response to FA desaturation may help explain differential sensitivities to lipid desaturation and inform therapeutic strategies in cancer and regenerative contexts.

## Graphical Abstract

Fatty acid desaturation regulates stem cell self-renewal through coordinated changes in membrane biophysics and downstream signaling. Alterations in fatty acyl chain desaturation modify key physicochemical properties of cellular membranes, including fluidity, thickness, and curvature, thereby influencing membrane protein activity and organelle homeostasis. These membrane-driven changes propagate through intracellular pathways, promoting endoplasmic reticulum stress responses and modulating inter- and intracellular signaling networks. Together, desaturation-dependent lipid remodeling establishes a regulatory framework that determines the balance between stem cell self-renewal and differentiation.

**Figure:**
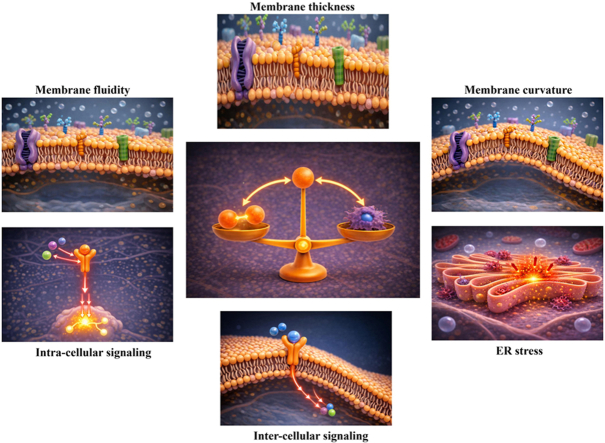

Cells with high differentiation potential, namely stem cells, have garnered significant scientific attention in recent years due to their pivotal roles in embryonic development, tissue homeostasis, and pathogenesis as well as their promise in regenerative medicine. Emerging evidence indicates that metabolic reprogramming plays a central role in governing the balance between self-renewal and differentiation in these cells.

Lipid metabolism constitutes a significant segment of the cellular metabolic network, playing essential roles in maintaining cell viability. Given the chemical versatility of lipids and their diverse biological activities, it is not surprising that specific lipid modifications can influence cellular viability in a context-dependent manner.

Numerous studies have demonstrated that certain cancer cells are particularly sensitive to perturbations in fatty acid (FA) desaturation, with inhibition of key enzymes such as stearoyl-CoA desaturase (SCD) resulting in decreased viability. Recent studies have highlighted a similar metabolic vulnerability to FA desaturation in cells with high differentiation potential, such as pluripotent stem cells (1) and cancer stem cells (2, 3, 4, 5, 6, 7, 8). Together, these findings suggest a potential link between a cell's differentiation potential and its reliance on SCD desaturation for maintaining cell survival.

However, our recent investigations into trophoblast stem cells (TSCs) metabolism reveal a contrasting scenario: these cells do not depend on SCD desaturation for survival; in fact, SCD desaturation may even be detrimental to these cells. These findings suggest a more nuanced relationship between FA desaturation and cell viability, indicating that the dependency on FA desaturation varies across cellular contexts with implications that may extend beyond stem cell systems.

Here, we discuss current perspectives on the mechanisms by which changes in membrane characteristics, induced by FA desaturation, regulate cell viability. Additionally, considering that FAs serve as precursors to numerous signaling molecules, we will briefly discuss the current understanding of FA desaturation impacts on cell signaling processes. Elucidating these mechanisms is critical for the understanding of the specific sensitivity of cells with high differentiation potential. As stem cell-specific mechanistic evidence remains limited, we frame somatic-cell literature as a mechanistic model.

## The Enzymes Involved in Lipid Desaturation

Lipid desaturation is a process that involves the removal of hydrogen atoms from FAs, leading to the formation of double bonds between carbon atoms within the fatty acyl chain. This biochemical modification is catalyzed by enzymes known as desaturases.

SCD, also known as delta-9 desaturase is an integral membrane protein located in the endoplasmic reticulum (ER), that facilitates the introduction of a double bond at the Δ9 position of saturated fatty acyl-CoAs. SCD prefers palmitoyl-CoA and stearoyl-CoA as substrates, converting them to palmitoleoyl-CoA and oleoyl-CoA, respectively (9, 10, 11, 12). In mammals, multiple isoforms of the Scd gene have been identified, including four isoforms in mice (Scd1–4) and two isoforms in humans (SCD1 and SCD5) (13).

The desaturation of FAs by SCD1 has been shown to support the survival of cancer cells across multiple tumor types (9, 13, 14, 15, 16, 17, 18, 19, 20, 21, 22), including hepatocellular carcinoma (23), liver cancer (2), ovarian cancer (24), glioblastoma (3), colon (4), and lung (25) cancer.

Recent studies have also highlighted a similar dependence on SCD activity in pluripotent stem cells (PSCs) (1), raising the possibility that the requirement for MUFAs may vary with cellular differentiation state. Accordingly, many solid-tumor cancer stem cell models exhibit sensitivity to SCD1 inhibition; however, this dependency is not universal (2, 3, 4, 5, 8, 26, 27). For example, in chronic myeloid leukemia stem cells, Scd1 has been reported to exert a tumor-suppressive role, where its upregulation reduces leukemia stem cell survival (26). Taken together, these findings suggest that the relationship between differentiation potential and reliance on SCD-mediated desaturation for maintaining cellular viability is context dependent.

This highlights the need for a deeper mechanistic understanding of how FA desaturation and, in particular, SCD regulates cell viability across different cellular states.

The biosynthesis of polyunsaturated fatty acids (PUFAs), which contain multiple double bonds, involves a series of consecutive desaturation and chain elongation reactions. These processes occur primarily within the endoplasmic reticulum (ER) (28). Mammals, including humans, lack the Δ12 desaturase enzyme required to introduce double bonds at the Δ12 position of FAs, a critical step in the biosynthesis of PUFAs. Consequently, these essential FAs must be obtained through dietary intake (28, 29). PUFAs with double bonds in the omega-6 (n-6) position are synthesized from linoleic acid (18:2, n-6), while PUFAs with double bonds in the omega-3 (n-3) position are synthesized from α-linolenic acid (18:3, n-3). Both linoleic acid and α-linolenic acid are essential FAs that humans must obtain through their diet (30).

The Δ6 desaturase (D6D), encoded by FADS2, introduces a double bond at the Δ6 position, converting linoleic acid (18:2n-6) to γ-linolenic acid (18:3n-6). This product is then elongated to dihomo-γ-linolenic acid (20:3n-6), which is subsequently desaturated by the Δ5 desaturase (D5D), encoded by FADS1, to form arachidonic acid (20:4n-6). In the corresponding n-3 pathway, α-linolenic acid (18:3n-3) is converted to stearidonic acid (18:4n-3), which can be elongated to eicosatetraenoic acid (20:4n-3) (28, 30, 31).

## Metabolic Vulnerability to SCD1 is Associated with a High Differentiation Potential

The foundational concept of 'stem cell' is attributed to the pioneering work of James E. Till and Ernest A. McCulloch in the 1960s, demonstrating that colonies originated from progenitor cells possessing two critical properties: (1) the capacity to differentiate into multiple cell lineages, and (2) the ability to self-renew (32).

Stem cells are thus defined by both their potential to differentiate and their self-renewal capacity (33, 34, 35). PSCs possess the capacity to differentiate into any cell type of the adult organism, encompassing derivatives of all three germ layers: endoderm, mesoderm, and ectoderm. This remarkable potential enables PSCs to generate a diverse array of specialized cells (36). Pluripotency can be inherent, as observed in ESCs derived from the inner cell mass of the blastocyst, or acquired through genetic reprogramming, resulting in induced pluripotent stem cells (iPSCs) (37). Interestingly, PSCs exhibit notable similarities to cells with oncogenic transformation (38, 39), and specifically in their metabolic profiles (40). As such, they demonstrate a preference for glycolysis over oxidative phosphorylation, even in oxygen-rich environments, a phenomenon akin to the Warburg effect observed in cancer cells. This metabolic reprogramming supports rapid proliferation and biosynthetic demands. Furthermore, PSCs and subsets of cancer cells share self-renewal capacity and exhibit overlapping gene expression programs linked to pluripotency, stemness, and tumorigenesis. These parallels suggest that mechanisms governing pluripotency and oncogenic transformation may converge, highlighting the intricate relationship between stem cell biology and cancer pathophysiology.

The selective vulnerability of PSCs to FA desaturation has been unveiled by high-throughput screenings, which identified PluriSIn #1, an SCD1 inhibitor, inducing ER stress and apoptosis in human PSCs (hPSCs) (1).

In fact, comparative analyses revealed that hPSCs exhibit heightened sensitivity to SCD1 inhibition relative to various cancer cell lines, including neuroblastoma, hepatocarcinoma, cervical carcinoma (HeLa), and teratocarcinoma. Notably, this sensitivity persists even when hPSCs are cultured in basic fibroblast growth factor (bFGF)-depleted media, indicating that mere cell cycle exit does not confer resistance to SCD1 inhibition. This observation suggests a correlation between differentiation potential and susceptibility to FA desaturation blockade (27).

Further supporting this notion, transformed undifferentiated human primary skin fibroblasts, reprogrammed to acquire self-renewal and differentiation capacities, demonstrate significant sensitivity to the SCD1 inhibitors PluriSIn #1 and A939572. In contrast, their telomerase reverse transcriptase (hTERT)-immortalized counterparts exhibit minimal response (41). The cytotoxicity induced by SCD1 inhibition is specifically attributable to MUFA depletion, as evidenced by the rescue of cell viability upon oleate supplementation (27).

The vulnerability of stem cells to FA desaturation extends to non-pluripotent stem cell populations. Consistent with the concept that MUFA dependency is a hallmark of highly potent cellular states, numerous studies have demonstrated that cancer stem cells exhibit pronounced sensitivity to MUFA depletion (2, 3, 4, 5, 7, 8, 26, 27, 42, 43, 44, 45, 46, 47). Multipotent human mesenchymal stem cells (MSCs) are susceptible to palmitate-induced cytotoxicity, whereas oleate not only lacks toxicity but also offers protection against lipotoxic stress (47).

Together, these findings highlight the essential role of FA desaturation in supporting the viability of cells with high differentiation potential. They suggest a compelling interpretation: Such cells are particularly vulnerable to MUFA deficiency and exhibit pronounced sensitivity to SCD1 inhibition.

Our recent investigations have emphasized how desaturation requirements are cell-type specific and revealed a striking divergence in the lipid metabolic profiles of mouse ESCs and their extra-embryonic counterparts, TSCs. While ESCs exhibit a dependency on MUFAs for maintaining cell viability, TSCs display a contrasting sensitivity, with MUFAs exerting a toxic effect. This dichotomy is further underscored by the expression of distinct Scd1 protein variants: ESCs predominantly express a ∼35-kDa variant and a low MUFA content, whereas TSCs express a ∼70-kDa variant and a much higher MUFA content. These differences in Scd1 expression and MUFA levels highlight significant disparities in the lipid metabolism between these two cell types (48). In ESCs, the addition of oleic acid to the culture medium is essential for maintaining cell viability. In contrast, oleic acid supplementation is detrimental to TSCs.

The ∼70-kDa variant was previously observed and attributed to a protein dimer (49, 50); however, the protein sequences and structures of the Scd1 variants should be further elucidated.

Defining how these variants are differentially produced and regulated, processed, or localized, and how they impact enzymatic activity and lipid flux, will be essential for understanding how lipid metabolic programs are tailored to specific developmental contexts.

**Fig. 1:**
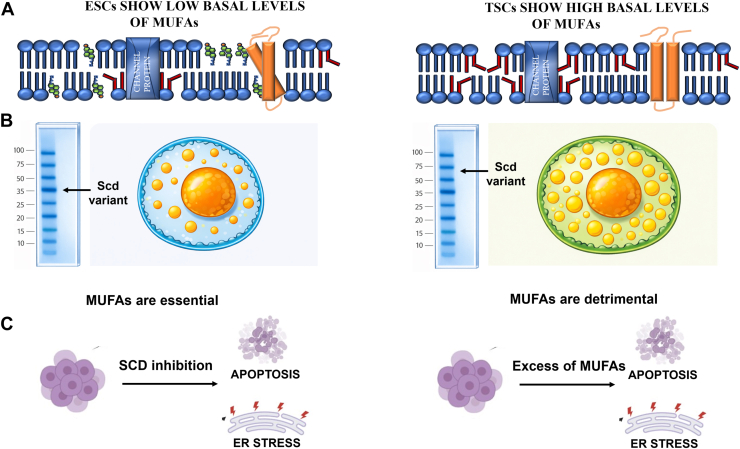
**Opposing dependencies on fatty acid desaturation in embryonic and trophoblast stem cells imply to a “desaturation window”.** (A) Embryonic stem cells (ESCs) exhibit low basal levels of monounsaturated fatty acids (MUFAs), whereas trophoblast stem cells (TSCs), their extra-embryonic counterparts, display high basal levels of MUFAs. (B) These differences are associated with the expression of distinct stearoyl-CoA desaturase (SCD) variants. (C) In ESCs, reduced FA desaturation disrupts membrane biophysical properties, induces endoplasmic reticulum (ER) stress, and leads to apoptosis, indicating a requirement for MUFAs. In contrast, excess MUFAs in TSCs induce ER stress and apoptosis, indicating that elevated MUFA levels are detrimental. These lineage-specific responses are consistent with a cell-type-specific “desaturation window” that governs membrane homeostasis, stress signaling, and the balance between stem cell maintenance and differentiation.

Figure 1 shows the differences in the sensitivity of blastocyst cell types (ESCs vs. TSCs) to mono-desaturation of FAs, as prominent examples to cell types with extreme opposite desaturation requirements.

The findings in this study suggest that the balance of FA desaturation may play a role in regulating the self-renewal and differentiation of blastocyst cells. They support a 'desaturation window' model, whereby deviations (excess saturation or excess desaturation) disrupt membrane homeostasis and trigger stress signaling pathways that alter stem cell fate. By this concept, cells with lower SCD activity and lower ratio of MUFA/saturated fatty acids (SFA) are addicted to MUFAs, whereas cells with higher SCD activity and MUFA/SFA ratio may even be sensitive to them. Understanding this balance is crucial, as it involves alterations in the biophysical properties of cellular membranes and the formation of bioactive lipid species that influence cell fate decisions. The expression of distinct Scd1 protein variants in ESCs and TSCs suggests divergent regulatory mechanisms governing lipid desaturation in the early embryo cell types.

Further studies are needed to elucidate how variations in SCD1 expression and MUFA levels orchestrate the metabolic and functional distinctions between cells in an embryo or an adult organism. Elucidating these mechanisms will enhance our understanding of how lipid metabolism influences stem cell fate decisions and developmental or pathological processes.

As a first step toward such understanding, and aligned with multiple other reports, our studies suggest that MUFA-dependent effects on membrane properties often make a major contribution to viability, whereas PUFA remodeling may more strongly influence differentiation-related phenotypes.

## FA Desaturation Impacts Cellular Membranes

Lipids constitute the primary structural components of biological membranes, and the desaturation of FAs within these lipids significantly influences membrane biophysical properties, including fluidity, intrinsic curvature, and thickness. These alterations, in turn, critically affect secondary characteristics such as membrane protein function, susceptibility to oxidative stress, and signal transduction pathways.

## FA Desaturation Alters Membrane Fluidity

Biological membranes exhibit distinct phase behaviors that are highly dependent on temperature, primarily transitioning between the gel phase and the liquid-crystalline phase. At lower temperatures, membranes adopt the gel phase, characterized by tightly packed, ordered lipid molecules resulting in a rigid structure. As temperature increases, membranes transition to the liquid-crystalline phase, where lipid molecules are more loosely packed and exhibit greater mobility, conferring fluidity to the membrane (51).

The critical regulation of membrane fluidity by FA desaturation is well-established and has been demonstrated throughout several animal kingdoms (52, 53, 54, 55, 56, 57). The introduction of double bonds into the acyl chains of membrane lipids induces kinks in the FA chains. These structural alterations disrupt the orderly packing of lipids, thereby enhancing membrane fluidity (58, 59, 60, 61). Notably, the transition from saturated lipid species to monounsaturated species results in a more pronounced decrease in transition temperature compared with the addition of subsequent double bonds (51). This observation underscores the pivotal role of MUFAs and SCD in modulating membrane fluidity and, consequently, cell viability. The impact of PUFAs on membrane fluidity is more complex, with varying effects depending on the specific PUFA species and the position of the double bonds. Generally, cis double bonds at the Δ9 position are more effective in enhancing membrane fluidity than those at the Δ6 position (62).

Membrane fluidity profoundly influences various cellular functions and viability. Alterations in membrane fluidity can affect permeability, protein function, signal transduction, membrane transport, and cell–cell interactions. We will discuss these in following sections. Comprehensive reviews on the regulation of cell viability and function by membrane fluidity provide further insights into these mechanisms (63, 64, 65, 66, 67, 68, 69).

## Mechanisms of Regulation of Membrane Fluidity

Membrane fluidity is a highly regulated property governed by multiple interrelated factors. A key determinant of fluidity is the ratio of saturated SFAs to MUFAs, which is largely controlled by the activity of SCD. The regulation of SCD activity has been extensively reviewed elsewhere (10, 70, 71, 72). In addition to SCD-mediated desaturation, several other factors critically influence membrane fluidity. These include the length of the fatty acyl chains, ambient temperature, and the presence of cholesterol within the membrane. Each of these parameters modulates the biophysical properties of the lipid bilayer, either by altering lipid packing density, modifying membrane thickness, or affecting phase behavior. We briefly outline the contribution of these variables to membrane fluidity, to provide a more comprehensive view of the regulatory landscape governing membrane physical properties, critically regulated by FA desaturation.

### Chain length

Increasing acyl chain length generally enhances lipid packing, increases acyl chain order, and raises the melting temperature; however, its effect depends on the degree of lipid desaturation. In saturated phospholipids, longer acyl chains strengthen van der Waals interactions between adjacent lipids, promoting tighter packing and reducing membrane fluidity. In unsaturated phospholipids, the presence of cis double bonds introduces kinks in the acyl chains that disrupt packing and increase conformational flexibility and disorder. As a result, the relationship between acyl chain length and membrane fluidity is not straightforward and cannot be interpreted independently of unsaturation. Moreover, “fluidity” itself is an insufficient descriptor of these effects (73, 74, 75).

### Temperature

The phase behavior of phospholipid bilayers is inherently temperature-dependent, with phase transitions from gel to liquid-crystalline states influenced by ambient conditions. In unicellular organisms, modulation of membrane fluidity via FA desaturation serves as a pivotal adaptive mechanism to counteract environmental temperature fluctuations. In *Saccharomyces cerevisiae*, the Δ9 FA desaturase gene, OLE1, encodes an enzyme responsible for converting saturated FAs to MUFAs, thereby modulating membrane fluidity. Expression of OLE1 is tightly regulated and can be transiently activated in response to low temperatures, facilitating adaptation to cooler environments (76, 77). Similarly, in the cyanobacterium *Synechocystis*, three of the four desaturase genes, namely *desA*, *desB,* and *desD* are cold-inducible. These genes are upregulated following a temperature downshift, enhancing the desaturation of membrane lipids to maintain appropriate fluidity under cold stress (64). In *Bacillus subtilis*, the Δ5 desaturase (D5D) is encoded by the *des* gene, whose expression is governed by a two-component regulatory system comprising the histidine kinase DesK and the response regulator DesR. Upon sensing decreased temperatures, DesK undergoes autophosphorylation and subsequently activates DesR, which induces *des* gene expression. The resulting desaturase introduces double bonds into FAs, thereby increasing membrane fluidity to adapt to colder conditions (76).

### Cholesterol

Cholesterol plays a pivotal role in modulating the fluidity of mammalian cell membranes. Early studies demonstrated that increasing the cholesterol-to-phospholipid ratio in erythrocyte membranes leads to a reduction in membrane fluidity (78). However, the influence of cholesterol is more nuanced and temperature-dependent. At elevated temperatures, cholesterol stabilizes the membrane by restraining the movement of phospholipid FA chains, thereby decreasing fluidity. Conversely, at lower temperatures, cholesterol prevents the FA chains from clustering together, thus maintaining membrane fluidity (79). In *Saccharomyces cerevisiae*, the interaction between saturated SFAs and ergosterol (the yeast equivalent of cholesterol) has been shown to induce ER stress. This stress response is linked to the accumulation of misfolded proteins, suggesting that the cholesterol-phospholipid interaction critically influences protein folding and cellular homeostasis (80) (more on FA desaturation and ER stress herein).

## FA Desaturation Alters Membrane Intrinsic Curvature

The structural characteristics of acyl chains significantly influence membrane curvature by altering phospholipid geometry (51). Double bonds (in particular, cis-configured double bonds) introduce kinks into hydrocarbon chains, causing phospholipids to adopt a wedge-shaped conformation that enhances membrane curvature. Additionally, a higher proportion of monounsaturated or unsaturated lipids tends to reduce the average acyl chain length within the membrane, further promoting curvature (81, 82). Notably, an increase in lipid saturation leads to the flattening of membranes, including those of the nuclear envelope and ER, into more planar and polygonal structures that are prone to fracture (83). Membrane curvature plays a crucial role in modulating protein conformation, mobility, and function (51). Alterations in curvature can also impact the dimerization of cholesterol (84). Maintaining balanced lipid saturation is therefore essential for preserving membrane physiological curvature and downstream cellular function.

## FA Desaturation Alters Membrane Thickness

The composition of FAs within lipid bilayers significantly influences membrane thickness (61, 81). A higher proportion of unsaturated FAs leads to a reduction in bilayer thickness. Membrane thickness, in turn, affects the function of integral membrane proteins. Notable examples include the erythrocyte glucose transporter, rhodopsin, Ca^2+^-activated potassium channels, sarcoplasmic reticulum Ca^2+^-ATPase, Na^+^/K^+^-ATPase, cytochrome c oxidase, F_1_F_0_-ATP synthase, and various bacterial transporters such as the Leu-Na^+^ cotransporter in *Pseudomonas aeruginosa*, the Leu-H^+^ cotransporter in *Lactobacillus lactis*, the melibiose-cation cotransporter in *Escherichia coli*, and diacylglycerol kinase in *Escherichia coli* (85, 86).

Membrane thickness also plays a critical role in determining membrane permeability. As such, the permeability coefficients for water and neutral polar solutes decrease approximately five-fold as the acyl chain length increases from 14 to 24 carbon atoms. For protons and potassium ions, permeability decreases by two orders of magnitude when the chain length extends from 14 to 18 carbon atoms and plateaus with further elongation to 24 carbon atoms (87). Furthermore, cholesterol's behavior within the membrane is influenced by its dimerization, which is stringently regulated by a narrow window of membrane thickness (84). This dimerization can subsequently impact the function of membrane proteins and channels, notably stabilizing the inactivated state of inward-rectifier potassium channels (88).

**Fig. 2:**
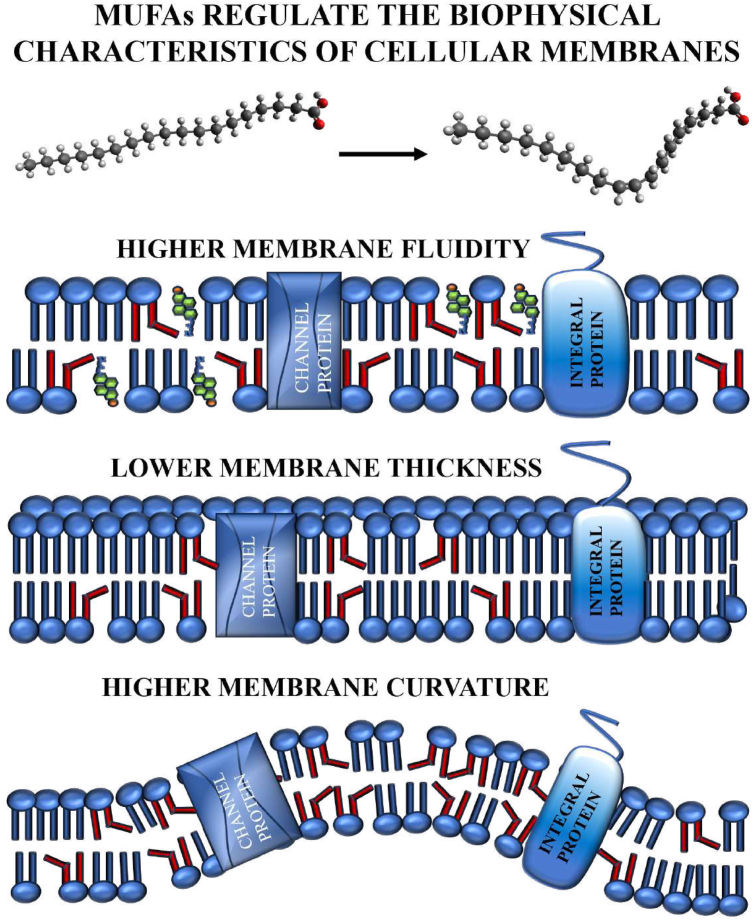
Fatty acid desaturation regulates stem cell self-renewal through membrane biophysical remodeling. Stearoyl-CoA desaturase 1 (SCD1) generates monounsaturated fatty acids (MUFAs) that reshape membrane lipid composition and biophysical properties of the lipid bilayer. Increased acyl chain unsaturation modulates membrane fluidity, thickness, and curvature, thereby influencing the conformation, organization, and activity of membrane proteins such as ion channels and receptor complexes. Through these biophysical effects, fatty acid desaturation links membrane lipid remodeling to signaling pathways that support stem cell identity and self-renewal.

Figure 2 depicts the major physicochemical alterations in cellular membranes conferred by FA desaturation.

## Endoplasmic Reticulum (ER) Homeostasis

The ER is a membranous organelle integral to protein processing and lipid metabolism (89). Stress within the ER triggers the unfolded protein response (UPR), a coordinated reaction aimed at managing the accumulation of misfolded proteins, and at restoring ER homeostasis. The desaturation of phospholipid acyl chains that induces biophysical alterations across cellular membranes has particularly significant functional impacts on the ER. Inositol-requiring enzyme 1 (IRE1) and protein kinase RNA-like endoplasmic reticulum kinase (PERK) are integral transmembrane sensors within the ER that detect perturbations in membrane phospholipid composition, particularly alterations in the saturation levels of lipid acyl chains. Such lipid bilayer stress can directly activate IRE1 and PERK independently of unfolded protein accumulation. Upon activation, these sensors initiate the UPR; if the stress persists, prolonged UPR activation may lead to apoptosis (90).

Membrane fluidity is crucial for ER function; disruptions can lead to protein misfolding and aggregation within the ER lumen, potentially activating the UPR and leading to apoptosis.

In fact, palmitate and stearate (but not oleate or linoleate) have been shown to induce ER stress and apoptosis in various cell types. In H4IIE liver cells, these SFAs triggered ER stress and apoptosis, accompanied by increased ceramide levels. However, inhibiting de novo ceramide synthesis did not prevent SFA-induced ER stress and apoptosis, suggesting that these effects are independent of ceramide accumulation (91). In meniscus cells, palmitate (but not oleate) induced the expression of C/EBP homologous protein (CHOP) and X-box binding protein 1 (XBP1), activated c-Jun N-terminal kinase (JNK), and increased caspase-3 expression, promoting apoptosis. Treatment with chemical chaperones or inhibitors of IRE1α signaling mitigated these effects, indicating the involvement of the UPR in palmitate-induced apoptosis (92). Knockdown of SCD1 in HeLa cells increased the proportion of SFAs in phospholipids, leading to UPR activation, as evidenced by elevated CHOP and glucose-regulated protein 78 (GRP78) mRNA levels and splicing of XBP1 mRNA. In hepatocellular carcinoma (HCC) cells with high expression of v-myc avian myelocytomatosis viral oncogene neuroblastoma-derived homolog (MYCN), increased levels of unsaturated FAs were observed. Inhibition of FA desaturation suppressed cell proliferation and induced ER stress-related signaling, indicating a link between lipid desaturation and ER stress in HCC cells (23). In human mesenchymal stromal cells and osteoblasts, palmitate was cytotoxic, whereas oleate was in fact protective against lipotoxicity. Activation of liver X receptors (LXRs) induced SCD1 expression, significantly reducing palmitate-induced cell mortality, caspase-3/7 activation, ER stress, and inflammation (47). In glioblastoma stem-like cells, depletion of oleic acid, impairment of SCD1 activity, or supplementation with palmitic acid exacerbated IRE1-mediated ER stress through the accumulation of SFAs, triggering apoptosis (3). Pancreatic β-cells exposed to palmitate under serum-free conditions exhibited significant apoptosis and increased protein levels of phosphorylated eukaryotic translation initiation factor 2α (eIF2α), activating transcription factor 4 (ATF4), XBP1, and CHOP (93). Palmitate- (but not oleate-) treated cells also showed altered distribution of ER chaperones and ER morphology. Chronic exposure to palmitate, but not oleate, induced UPR pathways, altered ER chaperone distribution, and contributed to pancreatic β-cell death, implicating ER stress in the development of type 2 diabetes (94).

These findings underscore the critical role of balanced lipid saturation in maintaining ER integrity and function. Disruptions in lipid composition, can lead to ER stress and activation of the UPR, potentially resulting in apoptosis and contributing to various pathologies.

## The Introduction of Additional Double Bonds into Fatty Acids (FAs) May Influence Membrane Fluidity and Endoplasmic Reticulum (ER) Homeostasis

While the desaturation of saturated fatty acids (SFAs) to monounsaturated fatty acids (MUFAs) is well established as a key regulator of membrane fluidity and ER function, the role of polyunsaturated fatty acids (PUFAs) in these processes remains less clearly defined (95). Emerging evidence indicates that further desaturation can modulate ER stress responses. In *Arabidopsis thaliana*, the FA desaturase 2 (FAD2) enzyme converts oleic acid to linoleic acid. Mutations in FAD2 reduce lipid poly-unsaturation and render plants hypersensitive to chemically induced ER stress. This indicates that FAD2-mediated poly-unsaturation is important for ER stress tolerance in plants (96). Nevertheless, the impact of PUFAs on ER stress depends on lipid composition and cellular environment. In apparent contrast to its expected protective effects, docosahexaenoic acid (DHA; 22:6 n-3) has been shown to induce ER stress and growth arrest in human colon cancer cells, suggesting a potential pro-apoptotic role in certain cancer contexts (97). Conversely, DHA has been observed to mitigate palmitate-induced ER stress in human L02 hepatoma cells by reducing the expression of lectin-like oxidized low-density lipoprotein receptor-1 (LOX-1), supporting its protective effects against SFA-induced lipotoxicity in hepatic cells (98).

In the central nervous system, omega-3 PUFAs, including DHA, exert neuroprotective effects by attenuating ER stress through the inhibition of histone deacetylase 3 (HDAC3), leading to reduced inflammation and enhanced neuronal survival (99).

Collectively, these findings indicate that the effects of PUFAs on ER homeostasis are not uniform but depend on both lipid structure and cellular context, including chain length, degree and position of unsaturation, and cell type–specific signaling pathways (100).

## FA Desaturation Impacts Mitochondrial Function

While the role of ER membrane rigidification in cell death has been extensively studied, alterations in FA desaturation and the consequent changes in membrane fluidity of other organelles, notably the mitochondria, also significantly influence cellular function and viability. Alterations in mitochondrial membrane lipid composition, particularly cardiolipin (CL) acyl remodeling driven by FA desaturation, directly influence respiratory chain organization and apoptotic susceptibility. In macrophages, the incorporation of FAs into CL, pivotal mitochondrial phospholipids, is regulated by FA desaturation and mitochondrial transport processes (101). Specifically, oleic acid (18:1) is directly incorporated into CL, whereas linoleic (18:2) and α-linolenic acids (18:3) undergo desaturation and elongation prior to incorporation. In contrast, γ-linolenic (18:3) and stearidonic acids (18:4) are less favorably incorporated into CL. These findings underscore the critical role of FA desaturation in determining CL composition and, consequently, mitochondrial function. HeLa cultivated in panserin 401 without lipids can only utilize FAs derived from their own de novo biosynthesis. Palmitoleic acid (16:1) and OA (18:1) made up 80% of the total CL composition, showing an apparent preference of monounsaturated long-chain FAs over saturated ones (102).

Maintaining appropriate mitochondrial membrane fluidity is essential for normal mitochondrial operations, including bioenergetic flux and the structural integrity of cristae membranes (103). Disruptions in membrane fluidity can lead to decreased membrane fluidity, adversely affecting mitochondrial function and potentially leading to cell death. Therefore, the regulation of FA desaturation and the resulting preservation of mitochondrial membrane fluidity are vital for cellular health and viability.

## Membrane Protein Function

The lipid environment plays a pivotal role in modulating membrane protein activity by influencing protein folding, trafficking, organization, and function, consequently affecting overall cell viability and function (86). Membrane fluidity, in particular, governs the lateral mobility, spatial arrangement, and conformational dynamics of integral membrane proteins, such as receptors, transporters, and enzymes, thereby modulating signal transduction processes, including receptor clustering and activation (104).

A comprehensive review of the relationship between membrane fluidity and protein function is beyond the scope of this article. Instead, we highlight selected findings. Resulting alterations in protein folding, conformation, trafficking, and organization directly influence the functional capacity of membrane proteins, thereby modulating the efficiency of downstream signal transduction pathways. These regulatory effects are briefly discussed in this section.

### Signal transduction

FA desaturation-induced alterations in membrane composition can activate key cellular signaling pathways. We highlight several notable examples.

### G protein-coupled receptor (GPCR) signaling

Owing to their biological significance and well-characterized structure, GPCRs, especially rhodopsin, the photoreceptive GPCR, offer a valuable model for studying the lipid-mediated regulation of membrane protein function.

DHA and other PUFAs can influence membrane protein function through both indirect biophysical effects and direct lipid-protein interactions. Studies using rhodopsin as a model GPCR have shown that DHA-rich phospholipids alter membrane properties such as fluidity and packing, as well as lateral pressure, thereby modulating receptor conformational equilibria and signaling activity. In addition, molecular dynamics and biophysical analyses suggest that DHA-containing lipids can interact directly with rhodopsin, influencing its structural dynamics and stabilization of specific functional states (105, 106). Computational simulations suggest that phospholipids containing one saturated stearic acid chain and one polyunsaturated DHA chain exhibit preferential orientation that maximizes the interaction between the DHA chain and the surface of membrane proteins (107). In membranes enriched with 22:5 n-6 FAs, phosphodiesterase activity was reduced by approximately 50% compared with membranes containing n-3 PUFAs, suggesting that the n-3 double bond configuration confers a unique advantage in optimizing early signal transduction events (108). In reconstituted membrane systems, a reduction in DHA acyl chain content impaired rhodopsin’s ability to adopt the active metarhodopsin II conformation and to effectively bind transducin, accompanied by a corresponding decrease in phosphodiesterase activity (109). Alterations in membrane fluidity modulate the function of the endocannabinoid system by regulating the signaling activity of the cannabinoid receptor type 1 (CB1), a G-protein-coupled receptor highly expressed in the brain (110). Alterations in plasma membrane fluidity have also been implicated in disrupting β-adrenergic receptor function and impairing G-protein coupling (111).

### Notable examples of non-GPCR signaling regulated by FA desaturation and membrane physical properties

The relationship between membrane fluidity and hormone receptor interactions with adenylate cyclase's catalytic subunit was established already in the 1970s (112). Further studies have linked membrane fluidity to the epidermal growth factor receptor (EGFR) (113), the lateral mobility of membrane proteins such as Ras (114), and eukaryotic mechanosensitive channels that are sensitive to PUFAs (115). As one more example, fluidization of leukocyte membranes induced by aliphatic alcohols was shown to enhance the affinity of the N-Formylmethionylleucylphenylalanine (fMLP) receptor, which was concomitant with a decrease in the ED50 for fMet-Leu-Phe and a 1.5-fold increase in the maximal migration distance of cells (116).

### Calcium signaling

Reduced platelet membrane fluidity has been correlated with enhanced calcium mobilization from intracellular storage pools and elevated levels of free intracellular calcium in the presence of procaine in diabetic platelets (117). In Alfalfa (*Medicago sativa*) plant cells, membrane fluidization inhibited the induction of a cold acclimatization-specific gene (cas30), as well as calcium influx and freezing tolerance at 4°C (118). Under controlled conditions, membrane thicknesses that facilitate free calcium ion transport across biomimetic polymer membranes were found to range from 10.7 to 13.4 nm. Thicker membranes reduce, but not completely block transport (119). Intriguingly, the precise subcellular location of arachidonic acid release plays a critical role in determining calcium signaling outcome (120).

### Eicosanoid signaling

Desaturation of FAs is a pivotal step in the biosynthesis of signaling molecules such as eicosanoids, including prostaglandins, thromboxanes, and leukotrienes. These lipid mediators, primarily derived from PUFAs, most prominently arachidonic acid, play crucial roles in signal transduction pathways (121). A substantial body of evidence (e.g. (122, 123)) highlights the involvement of eicosanoids in modulating cell viability. For example, prostaglandin E2 (PGE2) and its derivative PGA2 can bind to the pro-apoptotic protein Bax, inducing conformational changes that promote apoptosis (124). These prostaglandins also downregulate key survival pathways, including the phosphatidylinositol 3-kinase (PI3K)/protein kinase B (PKB/Akt) signaling cascade (125), and the nuclear factor kappa-light-chain-enhancer of activated B cells (NF-κB) signaling pathway (126). Similarly, thromboxane A2 has been shown to induce apoptosis in immature thymocytes via the thromboxane A2 (TXA2) receptor (127), inhibit the phosphorylation of Akt (128), and require both leukemia-associated Rho guanine nucleotide exchange factor (Lsc) and Rho kinase (ROCK) for apoptotic signaling (129). Conversely, inhibition of the leukotriene B4 (LTB4) signaling pathway induces apoptosis through suppression of extracellular signal-regulated kinase (ERK) activation in colon cancer cells (130), and leukotriene D4 (LTD4) enhances proliferation in intestinal epithelial cells (131).

Notably, lipid mediators derived from n-3 essential PUFAs, such as DHA and eicosapentaenoic acid (EPA), exhibit anti-inflammatory and pro-resolving properties. These effects are believed to result from competitive inhibition of arachidonic acid metabolism and a concomitant reduction in pro-inflammatory eicosanoid production (123, 132). Collectively, PUFA-derived lipid mediators serve as potent regulators of inflammation and apoptosis, with n-3 and n-6 PUFAs exerting distinct and, at times, opposing effects on cell viability.

### Nuclear receptor signaling

FAs and their metabolic derivatives serve as endogenous ligands for multiple nuclear receptors, thereby influencing key transcriptional programs (133, 134, 135, 136). Among these, the peroxisome proliferator-activated receptors (PPARs) constitute a prominent family of nuclear receptors that act as ligand-activated transcription factors. PPARs are central regulators of diverse cellular processes, including lipid metabolism, inflammation, proliferation, and apoptosis. Unsaturated FAs, particularly PUFAs, exhibit higher binding affinity for PPARs compared with saturated counterparts (137). Upon FA binding, PPARs undergo conformational changes that promote the recruitment of transcriptional coactivators, thereby facilitating the transcription of target genes implicated in the regulation of cell fate decisions, such as proliferation and programmed cell death (137, 138, 139).

Some PUFAs and PUFA-derived molecules, particularly DHA in certain systems, have been reported to interact with retinoid x receptors (RXRs), , another class of nuclear receptors that dimerize with PPARs and other nuclear receptor partners (140), although the identity of the physiologically dominant endogenous RXR ligand remains controversial (141, 142). Through these heterodimeric complexes, RXRs modulate the transcription of genes involved in either promoting or inhibiting apoptosis (143, 144, 145).

### Oncogenic signaling

In addition to transcriptional regulation, FA desaturation has been linked to the activation of oncogenic signaling pathways. Notably, desaturation enhances the activity of pathways such as Wnt/β-catenin and Hippo/YAP, both of which are critical in the regulation of cell proliferation, differentiation, and tumorigenesis (146, 147).

## Membrane Permeability

Membrane fluidity and thickness are key biophysical properties that regulate membrane permeability, thereby influencing the passive diffusion of small molecules and ions. Alterations in lipid composition can significantly modulate these properties. For example, increased cholesterol-to-phospholipid ratios in erythrocyte membranes have been associated with decreased membrane permeability and a concomitant reduction in red blood cell lifespan in vivo (78). The degree of fatty acid (FA) desaturation in membrane lipids differentially modulates permeability to small molecules. Specifically, FA desaturation has been identified as a critical regulator of osmotic water permeability, significantly enhancing water diffusion across the bilayer. In contrast, membrane permeability to protons appears to be only modestly influenced by lipid fluidity (148), suggesting distinct regulatory mechanisms governing the permeation of different molecular species.

Modulation of membrane FA composition influences transmembrane transport processes, including insulin-regulated glucose uptake and passive amino acid permeability. In adipocyte membranes exhibiting insulin-stimulated D-glucose transport, treatment with SFAs resulted in a reduction of transport activity, whereas non-activated controls were unaffected, implicating increased FA desaturation and bilayer fluidity in the regulation of insulin-dependent glucose uptake (149). Supporting this interpretation, exogenous oleate was found to enhance basal glucose uptake in isolated adipocytes independently of changes in the subcellular distribution of glucose transporters GLUT1 and GLUT4 (150). FA desaturation has also been implicated in passive nutrient transport, as demonstrated in *Lactococcus lactis*, where increased lipid desaturation significantly elevated membrane permeability to leucine (151).

## Autophagy

Autophagy is a fundamental catabolic process responsible for the degradation and recycling of cytoplasmic components via lysosomal pathways (152, 153, 154). The autophagosomal isolation membrane is characterized by a high content of unsaturated FAs, which is thought to contribute to its dynamic properties. Although significant progress has been made in understanding autophagy over the past two decades, the molecular mechanisms driving the biogenesis of the autophagic membrane are not well understood (153, 155). Nonetheless, current evidence supports a critical role for MUFAs in promoting membrane fluidity, thereby facilitating autophagosome formation at the ER and activating autophagy. Inhibition of SCD1 suppresses starvation-induced autophagy; this inhibition can be reversed by either SCD1 overexpression or supplementation with oleic acid (152).

## Oxidative Stress and Cell Death

When the production of reactive oxygen species (ROS) surpasses the cell's detoxification capacity, macromolecular damage, particularly to proteins and DNA, may occur, ultimately leading to cell death (156). Membrane lipids, particularly those enriched in PUFAs, are primary targets of ROS owing to their high susceptibility to peroxidation conferred by multiple double bonds. Lipid peroxidation induces both functional and structural alterations in membranes, potentially resulting in membrane destabilization and cell lysis (157). Oxidized PUFAs form lipid peroxides that decompose into a range of reactive intermediates (158). Notably, PUFA-induced lipid peroxidation has been implicated in DNA damage (158), and in the activation of multiple forms of regulated cell death, including ferroptosis (159), pyroptosis (160) necroptosis (161) ER stress response (see above), leading to autophagy-dependent death (162) and parthanatos (163, 164). Given this, a lower degree of FA poly-desaturation in membrane lipids may confer resistance to oxidative damage by reducing susceptibility to peroxidation (165, 166). Supporting this notion and reciprocally linking it to mono-desaturation, SCD1 expression has been shown to downregulate PUFA levels and suppress lipid ROS accumulation and ferroptosis in gastric cancer stem cells (46).

**Fig. 3:**
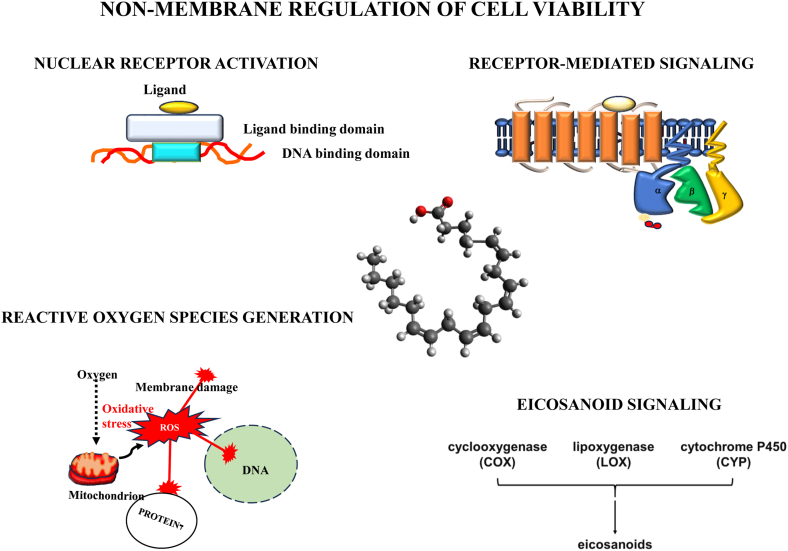
Lipid Desaturation and Cellular Viability: Conceptual Overview of Non-Membrane Mechanisms. Polyunsaturated fatty acids (PUFAs), schematically represented by arachidonic acid, act as ligands and precursors of lipid mediators that influence nuclear receptor activity, cellular signaling, and redox balance. Their metabolism via COX, LOX, and cytochrome P450 pathways generates eicosanoids, while also contributing to reactive oxygen species (ROS) production. These pathways collectively link lipid desaturation to cellular viability and stress responses. The figure is intended as a simplified conceptual overview.

Figure 3 summarizes several non-membrane mechanisms by which FA desaturation can influence cell viability, including nuclear receptor activation, receptor-mediated signaling, ROS generation, and eicosanoid signaling. These pathways represent indirect and context-dependent effects of FA desaturation that are generally less pronounced than the direct biophysical consequences of mono-desaturation driven changes in membrane properties.

## Regulating Membrane Biosynthesis

FA desaturation plays a critical role in lipid biosynthesis, as both the degree and positional distribution of double bonds within FA chains significantly influence the structural, functional, and physicochemical properties of lipid molecules. Moreover, the level of unsaturation affects the selective incorporation of FAs into specific lipid classes. For example, radiolabeling studies have demonstrated that [^14^C]-stearic acid is preferentially incorporated into triacylglycerols, whereas [^14^C]-oleic acid is predominantly incorporated into membrane phospholipids. This selective partitioning underscores the importance of MUFAs, such as oleic acid, in phospholipid biosynthesis and highlights their essential role in membrane formation and homeostasis (167).

## Cell Cycle

In our studies, we have not found significant FA desaturation-related changes in cell cycle in blastocyst stem cells. Still, rapidly proliferating cells must synthesize structural lipid macromolecules and remodel lipid signaling pathways to support cell division. Inhibition of SCD1 results in the accumulation of cells in the G0-G1 phase of the cell cycle, accompanied by a reduction in the S phase in benign PNT2 prostate epithelial cells (168), and arrests cell cycle progression in H460 lung cancer cells (25). Although the precise mechanisms underlying this regulation remain unclear, evidence suggests that Cyclin D1 and Cyclin-Dependent Kinase 6 (CDK6) may be involved (25).

## Concluding Remarks

FA desaturation emerges as a central regulator of cell viability, operating through an integrated network of biophysical and biochemical mechanisms. Across diverse cellular systems, particularly in stem cells, FA desaturation governs membrane properties, thereby influencing ER homeostasis, organelle function, and signaling pathways that collectively determine cell fate. An emerging concept arising from this body of work is the existence of a “desaturation window” in which cell viability is maintained within a defined range of lipid saturation. Deviations in either direction: excess saturation or excessive desaturation, disrupt membrane homeostasis, induce stress responses, and compromise survival. This window, and therefore cellular lipid requirements, is cell-type specific and context-dependent. Current evidence indicates that MUFA-driven alterations in membrane biophysical properties constitute a dominant mechanism. In contrast, PUFA remodeling appears to play more context-dependent roles, and are linked to differentiation and cellular signaling. A major outstanding challenge is to define the relative contributions and hierarchical organization of these mechanisms across different cellular states. Approaches combining targeted perturbations, lipidomic profiling, and functional rescue experiments will be essential to disentangle causal relationships within this highly interconnected network. Recent findings further suggest that FA desaturation is not merely permissive for cell survival but actively contributes to the fine-tuning of cell populations during development and disease. Elucidating how desaturation is dynamically regulated, and how it interfaces with cell state transitions, will be important for understanding stem cell biology and may reveal new therapeutic opportunities in cancer and regenerative medicine.

Collectively, available evidence positions FA desaturation as a fundamental regulator of cellular homeostasis, acting at the intersection of membrane biophysics, metabolic state, and cell fate determination.

## Data availability

All data generated or analyzed during this study are included in this published article and its supporting information files. Raw data is available upon request.

## Conflict of interest

The authors declare that they do not have any conflicts of interest with the content of this article.
